# Torsemide is a More Appropriate Oral Loop Diuretic for Patients with Heart Failure: Commentary

**DOI:** 10.34067/KID.0000000000000442

**Published:** 2024-04-09

**Authors:** Steven G. Coca

**Affiliations:** Barbara T. Murphy Division of Nephrology, Department of Medicine, Icahn School of Medicine at Mount Sinai, New York, New York

**Keywords:** chronic heart failure, diuretics

Chronic heart failure (CHF) is associated with significant morbidity and mortality worldwide. Control of congestion is the main therapeutic goal, because of the symptoms associated with volume overload as well as the mediation of poor outcomes and hospitalizations. Thus, use of diuretics is and has been central to the management of patients with CHF.

In this issue of *Kidney360*, Drs. Macon and Ellison debate Drs. Lo and Rangaswami on the appropriateness of torsemide as the optimal oral loop diuretic in patients with CHF.^1,2^ The reason why this debate has merit is that despite a much better pharmacokinetic (PK) and pharmacodynamic (PD) profile of torsemide over the other loop diuretics (including excellent oral bioavailability, faster onset of action, and longer duration of action), torsemide is used in only approximately 10% of patients with heart failure.

Drs. Macon and Ellison argue that the PK/PD profile of torsemide along with data from observational studies and small randomized controlled trials favor torsemide over the most commonly used diuretic, furosemide.^1^ The outcomes assessed in these studies demonstrated lower mortality, lower heart failure readmissions, and improvement in heart failure symptoms with torsemide versus furosemide. Their arguments for torsemide, however, reach a stumbling block when they come to the findings from the long-awaited “Effect of Torsemide vs Furosemide After Discharge on All-Cause Mortality in Patients Hospitalized With Heart Failure” (TRANSFORM-HF) clinical trial in over 2800 patients with CHF, which failed to demonstrate differences in mortality, hospitalizations, or symptoms between torsemide and furosemide.^3^ Macon and Ellison blame the null findings from the TRANSFORM-HF trial on issues with internal validity, including the pragmatic trial design that entailed allowing clinicians to perform routine care and its reliance on central data collection rather than in-person study visits. Indeed, these are excellent points that could have biased results to the null (as Macon and Ellison argue) because of glaring holes in key data elements, such as lack of knowledge of loop diuretic status, many missing study visit records, complete lack of information from hospital records, and a large proportion of missing survey data on quality-of-life measures (over 50% were missing the Kansas City Cardiomyopathy Questionnaire and Patient Health Questionnaire-9 surveys at 12 months). Indeed, while deducing that the findings were potentially null because of type 2 error, Macon and Ellison point out the painfully obvious fact that only approximately 1/3rd of individuals assigned to torsemide were known to be taking the drug at 12 months. There was also some crossover in both arms from torsemide to furosemide and *vice versa* (7% and 4%, respectively). Thus, I can sympathize with the authors in their favoring of torsemide and giving a mulligan on the large trial data that emerged from TRANSFORM-HF.

In their counterargument, Lo and Rangaswami acknowledge the PD/PK profile and early clinical data on torsemide that make it potentially the more attractive agent in this population.^2^ As expected, however, they lean heavily on the largely null data from the TRANSFORM-HF trial.^3^ They then dive into the pragmatism of diuretics and argue somewhat counterintuitively that the once-daily dosing of torsemide is actually a detriment because it has less potential to be titrated on the basis of adequacy of natriuretic response and potential for sequential nephron blockade and augmentation of diuresis with proximal tubular agents, including acetazolamide and sodium glucose cotransporter-2 inhibitor (SGLT2i) (albeit the argument pivoted toward inpatient management of heart failure). In their final section, they make the argument that de-escalation of diuretics with guideline-directed medical therapy (GDMT) is more difficult with the long-acting PK profile of torsemide. With that, they conclude that torsemide is not the ideal loop diuretic in part because of limited titratability (up or down) and the lack of benefit on clinical outcomes.

Where does that leave us? Let me be the ultimate contrarian by stating that my conclusion about diuretic choice in this patient population, after reading both sides of the arguments presented, one in favor of torsemide by Macon and Ellison and the other against by Drs. Lo and Rangaswami, is largely moot! How can I be so flippant after the two elegant detailed arguments that each side presented? First, diuretics are some of the most titratable drugs (to the upside and to the downside) in patients with CHF. They are not “set it and forget it” type of agents like some of the pillars of GDMT. Heart failure is a very heterogeneous syndrome, and the congestion associated with it is time-varying on an individual level. Thus, I almost see diuretics in the population as an “*n* of 1” type scenario, in which diuretic dosing is malleable and customizable on an individual basis depending on the myriad of factors that weigh into the decision for diuretic regimens (need for acute versus chronic volume control, serum potassium, history of efficacy or lack thereof on one loop versus the other, desire for fine tuning of natriuresis versus once daily dosing, *etc.*). What can be concluded from the clinical trial data, including from TRANSFORM-HF, is that it is unlikely that one loop diuretic is markedly better than the other agent in large populations of individuals with CHF, although it is likely that torsemide is noninferior to the other loop diuretics. Thus, if the once-daily dosing makes sense in a given patient, then evidence would not suggest that torsemide is a worse agent in most scenarios.

Second, as alluded to in the penultimate paragraph by Lo and Rangaswami, with the strong evidence for GDMT in the current era, the key for short and long-term clinical benefits is to initiate and continue GDMT therapies (*β*-blockers, angiotensin receptor/neprilysin inhibitors, SGLT2i, and mineralocorticoid antagonists).^4,5^ The diuretic decisions almost become secondary once the four pillars of GDMT are in place. To wit, only 8% of the TRANSFORM-HF population were even on SGLT2i. This is important because *post hoc* analyses of some of the landmark SGLT2i trials in heart failure have shown that randomization to SGLT2i results in decreased likelihood of diuretic dose escalation and an increased likelihood of diuretic de-escalation.^6,7^ Thus, the epidemic in heart failure is not going to be altered by changing the predominant loop diuretic from furosemide to torsemide. Instead, any and all efforts should go to trying to optimize the four pillars of GDMT, which still suffers from far too much therapeutic inertia (<10%–20% are on full GDMT), especially among women with HFrEF.^8,9^ While a lofty aspiration, it is my hope that if the clinical community were to achieve a much more ubiquitous implementation of GDMT in heart failure in the coming years, future generations of clinicians and scientists may scoff at the need to consider such frivolous questions as which loop diuretic is better than another.
